# Interleukin-10 exhibit dose-dependent effects on macrophage phenotypes and cardiac remodeling after myocardial infarction

**DOI:** 10.3389/fphys.2024.1481460

**Published:** 2025-01-15

**Authors:** Jing J. Zhang, Rodrigue Rizk, Xiaoping Li, Brandon G. Lee, Mason L. Matthies, Kennedy A. Bietz, Kang Kim, Johnny Huard, Yadong Wang, William C. W. Chen

**Affiliations:** ^1^ Division of Basic Biomedical Sciences, Sanford School of Medicine, University of South Dakota, Vermillion, SD, United States; ^2^ Department of Computer Science, University of South Dakota, Vermillion, SD, United States; ^3^ Department of Bioengineering, Swanson School of Engineering, University of Pittsburgh, Pittsburgh, PA, United States; ^4^ Center for Ultrasound Molecular Imaging and Therapeutics, Department of Medicine, University of Pittsburgh School of Medicine, Pittsburgh, PA, United States; ^5^ McGowan Institute for Regenerative Medicine, University of Pittsburgh, Pittsburgh, PA, United States; ^6^ The Linda & Mitch Center for Regenerative and Personalized Medicine, Steadman Philippon Research Institute, Vail, CO, United States; ^7^ The Biofoundry, Department of Biomedical Engineering, Cornell University, Ithaca, NY, United States

**Keywords:** interleukin-10, ischemic heart disease, myocardial infarction, immunomodulation, macrophage phenotype, cardiac remodeling

## Abstract

**Introduction:**

Interleukin-10 (IL-10) is a potent immunomodulatory cytokine widely explored as a therapeutic agent for diseases, including myocardial infarction (MI). High-dose IL-10 treatment may not achieve expected outcomes, raising the question of whether IL-10 has dose-dependency, or even uncharted side-effects from overdosing. We hypothesized that IL-10 has dose-dependent effects on macrophage (Mφ) phenotypic transition and cardiac remodeling after MI.

**Methods:**

Using RAW264.7 monocyte models, we examined whether administering differential doses of exogenous IL-10 (0–1,000 ng/mL) perturbs classic M1 (pro-inflammatory) and M2 (anti-inflammatory) phenotypes of polarized Mφ or even alters the phenotypic transition of prospective M1 and M2 polarization. We then investigated the impact of single intramyocardial IL-10 administration on cardiac function, structure, and inflammation post-MI, using a mouse MI model.

**Results:**

Compared with 0-ng/mL control, 250-ng/mL IL-10 had the strongest overall effects in decreasing M1 and increasing M2 phenotypes on polarized Mφ while ≥500-ng/mL IL-10 dampened M1 polarization and augmented native IL-10 secretion more effectively than low doses *in vitro*. Echocardiography revealed that the 250-ng group had consistently higher contractile function and lower left ventricular (LV) dilatation than the saline control over 6 weeks while ≥1,000-ng groups exhibited transient lower LV ejection fraction at 5 days post-MI *in vivo*. Moreover, different doses of IL-10 differentially modulated myocardial gene expression, phagocytic cell infiltration at the infarct, LV fibrosis, and revascularization post-MI, with some, but not all, doses exerting beneficial effects.

**Discussion:**

Our study suggested that IL-10 has an effective dose range on Mφ phenotypes, and intramyocardial IL-10 treatment may trigger cardioprotective or unwanted effects post-MI in a dose-dependent manner.

## Introduction

In 2023, cardiovascular disease (CVD) caused roughly 20.5 million deaths worldwide, representing an 18.5% increase in the CVD-associated death rate compared to that of 2010 (Tsao et al., 2023). The role of tissue inflammatory responses in cardiac remodeling and functional recovery following myocardial damage has been widely investigated in recent years (Epelman et al., 2015; Frangogiannis, 2015; Santos-Zas et al., 2018; Halade and Lee, 2022). The early-phase inflammatory responses are primarily initiated and propagated by the innate immune system soon after myocardial injury and typically involve multiple secretory cytokines that mediate a variety of pathophysiological activities within and around the damaged tissue, such as cell death, myocardial fibrosis, metabolic homeostasis, and tissue regeneration (Halade and Lee, 2022; Frangogiannis, 2014; Pluijmert et al., 2021). For example, the expression of tumor necrosis factor (TNF)-α, a critical pro-inflammatory cytokine, significantly increases in the failing myocardium (Schumacher and Naga Prasad, 2018). Interleukin-6 (IL-6) is a versatile cytokine that is notably elevated and primarily activates pro-inflammatory responses after acute myocardial infarction (AMI) (Anderson et al., 2013). On the other hand, interleukin-10 (IL-10) is a pleiotropic cytokine that exhibits broad immunoregulatory and anti-inflammatory activities (Iyer and Cheng, 2012; Saraiva et al., 2020; Carlini et al., 2023; York et al., 2024). IL-10 not only inhibits the expression of the essential pro-inflammatory cytokines, including TNF-α, interleukin-1β (IL-1β), and IL-6 but also reduces the production of macrophage (Mφ)-derived reactive oxygen species (ROS) and nitric oxide (NO) (Saraiva et al., 2020).

In the cardiac milieu, the decrease of IL-10 expression over time following MI has been associated with reduced long-term cardiac function (Kaur et al., 2006). The increased plasma levels of anti-inflammatory mediators following intravenous immunoglobulin administration, including IL-10, notably correlated with augmented left ventricle (LV) contractile function in patients with congestive heart failure (Gullestad et al., 2001). Moreover, daily subcutaneous administrations of recombinant human IL-10 (rhIL-10, 10 μg/animal/day) significantly improved the survival of mice suffering from viral myocarditis, attenuated myocardial lesions, and suppressed pro-inflammatory cytokine production (Nishio et al., 1999). In a rat model of AMI, subcutaneous injections of rhIL-10 (75 μg/kg/day) continuously for 4 weeks resulted in significantly enhanced LV function, decreased serum levels of pro-inflammatory cytokines/chemokines such as TNF-α, IL-6 and monocyte chemoattractant protein-1 (MCP-1), and reduced myocardial macrophage infiltration (Stumpf et al., 2008). It was further demonstrated that systemic administrations of mouse IL-10 (50 μg/kg, 5 times post-MI) in a mouse AMI model significantly reduced inflammatory responses, improved LV function, and mitigated cardiac remodeling (Krishnamurthy et al., 2009). Two major mechanistic pathways haven been identified to contribute to the IL-10-mediated therapeutic benefits: inhibiting cardiac fibrosis through human antigen R/matrix metallopeptidase 9 (HuR/MMP-9) suppression and increased myocardial capillary density through signal transducer and activator of transcription 3 (STAT3) activation (Krishnamurthy et al., 2009; Haghikia et al., 2014). Thus, IL-10 treatment has a potential to modulate myocardial inflammatory responses and benefit cardiac structural and functional recovery post-MI (Jung et al., 2017; Tesoro et al., 2022).

Mφ are versatile innate immune cells that play key roles in the initiation, propagation, and resolution of myocardial inflammation after MI (Bruton et al., 2022; Gao et al., 2021; Kim et al., 2021). Both resident and recruited Mφ contribute to the clearance of the dead cells and cellular debris, the initiation of the repair process, and the modulation of the inflammatory responses (Zhang et al., 2021; Wu and Lu, 2019; Salina et al., 2022). These Mφ are classically categorized into two broad functional groups with distinct phenotypes: M1 or inflammatory Mφ that are pro-inflammatory and primarily contribute to the initiation and propagation of the post-injury inflammatory responses, and M2 or noninflammatory Mφ that largely participate in the resolution of inflammation as well as tissue repair and recovery (Yunna et al., 2020; Cheng and Rong, 2018; Ni et al., 2021; Jian et al., 2023). Failure of the Mφ participation in the post-injury immune responses resulted in impaired healing and chronic inflammation (Zuo et al., 2023; Krzyszczyk et al., 2018; Ganesh and Ramkumar, 2020). IL-10 has been shown to play a pivotal role in limiting the injury-induced inflammatory responses and facilitating the Mφ phenotypic switch from inflammatory to anti-inflammatory (Saraiva and O'Garra, 2010; Ip et al., 2017). IL-10 treatment can modulate macrophage polarization, reducing their secretion of pro-inflammatory cytokines and enhancing their contribution to cardiac tissue repair post-MI (Krishnamurthy et al., 2009; Jung et al., 2017). Nevertheless, despite its promise, the therapeutic potential of direct intramyocardial administration of IL-10 remains untested.

Furthermore, the large, repeated therapeutic doses of IL-10 required for the subcutaneous or systemic administration in prior studies not only increase the risks of potential unwanted or off-target effects but also substantially raise the cost of the treatment (Asadullah et al., 2003). For example, dramatically elevated serum IL-10 in patients has been reported to play pro-inflammatory and immune-activating roles in COVID-19 pathogenesis, suggesting the unwanted effects of overly high circulating IL-10 concentrations (Lu et al., 2021; Zhao et al., 2020; Han et al., 2020). To facilitate preclinical and clinical translation of IL10-based therapeutics for ischemic heart disease, in this study, we addressed the knowledge gap between the therapeutic dosage of IL-10, immune modulation, cardiac repair, and possible unwanted effects. We hypothesized that the Mφ phenotype polarization could be programmed with appropriate doses of IL-10 to favor tissue repair and that early localized administration of IL-10 into the ischemic myocardium could ameliorate myocardial inflammation, cardiac remodeling, and LV dysfunction, leading to enhanced cardiac structural and functional recovery post-MI. To test our hypotheses, the current study explored dose-dependent effects of IL-10 on programming classic macrophage phenotypes and assessed the therapeutic potential of an early, single-dose intramyocardial injection of IL-10 in a mouse MI model.

## Materials and methods

### Induction of macrophage polarization *in vitro*

We used RAW264.7 murine monocyte/Mφ cell line as a model for evaluating the polarization of Mφ phenotypes. To induce M1 polarization, RAW264.7 cells were treated with 20 ng/mL recombinant murine interferon-gamma (mIFNγ; 315-05, PeproTech, Cranbury, NJ, United States) for 24 h. To induce M2 polarization, RAW264.7 cells were treated with 20 ng/mL recombinant murine interleukin-4 (mIL-4; 214-14, PeproTech) for 48 h. Cellular morphology was observed and imaged daily post-induction with the EVOS M7000 microscope (ThermoFisher Scientific, Waltham, MA, United States). Flow cytometry was used to confirm the outcome of the induction. For M1 polarization, we labeled cells with a primary rat anti-mouse CD86 antibody conjugated with Alexa Fluor 488 (105,017, BioLegend, San Diego, CA, United States). For M2 polarization, we labeled cells with a primary rat anti-mouse CD206 (MMR) antibody conjugated with Alexa Fluor 488 (141,709, BioLegend, San Diego, CA, United States).

### Dose-dependent effects of IL-10 on macrophage phenotypes *in vitro*

To examine the phenotypic effects of differential dosage of IL-10 on the classic M1/M2 polarization of Mφ *in vitro*, we used rhIL-10 (200-10, PeproTech). We chose rhIL-10 because it is functionally active on both murine and human cells, according to the manufacturer’s instructions and previous literature (Nishio et al., 1999; Stumpf et al., 2008). We implemented two distinct experimental protocols to investigate the impact of IL-10 on 1) phenotypes of established Mφ polarization or 2) prospective programming of Mφ polarization. In protocol #1, naïve RAW 264.7 cells were first induced with mIFNγ and mIL-4 for 24 and 48 h to establish the classic M1 and M2 phenotypes, respectively, followed by individual treatment with different doses of IL-10 (0, 25, 100, 250, 500, and 1,000 ng/mL) for 24 h. In protocol #2, naïve RAW 264.7 cells were co-stimulated with a phenotype-polarizing agent (mIFNγ for M1 or mIL-4 for M2) in conjunction with one of the six IL-10 doses (0, 25, 100, 250, 500, or 1,000 ng/mL) for 36 h. Cellular morphology was observed and imaged daily with the EVOS M7000 microscope. Flow cytometry and cytokine secretion were used to quantify the phenotypic and functional outcomes of these experiments, respectively.

### Flow cytometry

Cells labeled with fluorescence-conjugated primary antibodies were analyzed with an Accuri C6 flow cytometer, equipped with 405, 488, and 561 nm lasers (BD Biosciences, San Jose, CA, United States). Ten thousand events per sample were collected for analysis. Sphero rainbow calibration particles with eight peaks (RCP-30-5A, Spherotech, Lake Forest, IL, United States) were used for instrument normalization and MEFL calculation. Briefly, RAW264.7 cells were detached by scraping, washed and resuspended in phosphate-buffered saline (PBS), and quantified using a Countess 3 FL automatic hemocytometer (AMQAF 2000, Thermo Fisher Scientific Cellular Analysis, Eugene, OR, United States). The cell concentration was then adjusted to 1 × 10^6^ cells/mL before incubating them for 20 min on ice with fluorescence-conjugated primary antibodies against specific cell lineage markers that were diluted in FACS buffer (PBS with 1% bovine serum albumin) and protected from light. Cells were then washed twice with PBS to remove excessive antibodies and resuspended in 500 μL of FACS buffer for analysis. For each sample, 10,000 events were collected to ensure statistical significance of the data. Flow cytometry data were analyzed and quantified with FlowJo (FlowJo LLC, Ashland, OR, United States) and represented in histograms (single parameter).

### Enzyme-Linked Immunosorbent Assay

Enzyme-Linked Immunosorbent Assay (ELISA) was performed to quantify the amounts of TNF-α and murine IL-10 (mIL-10) in the cell culture supernatants that were secreted by the induced RAW264.7 cells treated with different doses of IL-10. The sandwich ELISA development kits specific for capturing murine TNF-α (900-T54) and mIL-10 (900-T53) were used with ELISA TMB Buffer Kit (900-T00) (all from PeproTech) for the colorimetric detection in prepare 96-well standard ELISA microplates (Greiner Bio-One North America, Monroe, NC, United States). ELISA was performed following the manufacturer’s instructions. Absorbance readings at 450 nm were recorded with an EnSpire Multilabel Reader (Perkin Elmer, Shelton, CT, United States).

### Experimental animals

The Institutional Animal Care and Use Committee (IACUC) at the University of Pittsburgh (UPitt) and University of South Dakota (USD) approved the animal usage and surgical procedures performed in this study (UPitt IACUC Protocol 12010140 and USD IACUC Protocol 03-01-22-25D). A total of 73 male 10–12 weeks old BALB/cJ mice (Jackson Laboratory, Bar Harbor, ME, United States) were used for this study.

### Intramyocardial injection of IL-10 in acute myocardial infarction model

The same rhIL-10 employed *in vitro* was also used for *in vivo* studies. The induction of AMI and intramyocardial injections were performed as we previously described (Figure 3A) (Xiang et al., 2011; Chen et al., 2013; Chu et al., 2013) Briefly, after the induction of anesthesia with 4% isoflurane gas, mice were intubated and inhalationally anesthetized with 2% isoflurane gas throughout the surgery. MI was microscopically induced by permanent ligation of the left anterior descending (LAD) coronary artery. Mice were then randomly assigned to one of the six IL-10 treatment groups: 25, 100, 250, 500, 1,000, and 2000 ng of rhIL-10 (all diluted in 30 µL of sterile 0.9% saline). Ten minutes after the induction of MI, injections were microscopically performed at three sites of the ischemic myocardium (at the center and two borders of the infarct area, each receiving 10 µL solution). Control mice received 30 µL saline (Baxter Healthcare, Deerfield, IL, United States) at the same three sites, following the same procedures.

### Echocardiography

Echocardiographic studies were performed by a blinded investigator repeatedly before surgery and at 5 days, 2 weeks, and 6 weeks post-surgery to assess the cardiac function as described previously (Chen et al., 2013; Chu et al., 2013; Okada et al., 2012). Concisely, mice were initially anesthetized with 2% isoflurane gas and subsequently maintained at 1%–1.5% isoflurane gas throughout the echocardiographic study. Mice were then immobilized on a heated stage equipped with electrocardiography. Heart rate, respiratory rate, and body temperature were continuously monitored and maintained. Echocardiographic parameters were measured using high-frequency ultrasound scanners (Vevo 770 and 2,100 at the Center for Ultrasound Molecular Imaging and Therapeutics, University of Pittsburgh Medical Center, and Vevo 3,100 at the University of South Dakota (USD) Animal Resource Center; both from FUJIFILM VisualSonics Inc., Canada). End-systolic dimension (ESD) and end-diastolic dimensions (EDD) were determined from the short-axis images of the LV using M-mode. At least eight consecutive beats were measured. End-systolic area (ESA) and end-diastolic area (EDA) were measured from short-axis images of the LV using B-mode. Contractile parameters, including LV fractional shortening (LVFS) and LV ejection fraction (LVEF), were determined as previously described (Manning et al., 1994; Pollick et al., 1995; Wandt et al., 1999). Mice that were sacrificed for histological analyses or died prior to the end point of 6 weeks post-injection were excluded from the echocardiographic studies.

### Histological and immunohistochemical analyses

Animals were sacrificed at 6 weeks post-surgery. Hearts were harvested and arrested in diastole by immediately immersing in 1 M potassium chloride (KCl) and processed as previously reported (Xiang et al., 2011; Chen et al., 2013; Chu et al., 2013). Hearts were flash-frozen in 2-methylbutane (Sigma-Aldrich, St. Louis, MO, United States) that was pre-cooled in liquid nitrogen or dry ice. Frozen hearts were stored at −80°C and subsequently serially cryosectioned at 8-µm thickness from the apex to the ligation level (approximately 0.5 mm in length). Heart slices at 1-mm thickness were collected immediately below the ligation level for molecular analyses from randomly selected heart samples. Each series contains 18–24 heart sections, which are roughly 200 µm apart natively and collected on one glass slide. Sections were fixed in a pre-cooled (−20°C) mixture of methanol and acetone (1:1) for 5 min or in 4% paraformaldehyde for 8 min at room temperature (RT) immediately prior to staining (all from Sigma-Aldrich). For immunohistochemistry, non-specific antibody binding was blocked with 10% donkey or goat serum for 1–2 h at RT and, if necessary, with the Mouse-on-Mouse (M.O.M.) antibody staining kit (Vector Laboratories, Burlingame, CA, United States). For evaluation of chronic inflammation, sections were incubated overnight at 4°C with rat anti-mouse CD68 primary antibody (1:200, Abcam, Cambridge, MA, United States), followed by goat anti-rat-Alexa488 IgG (1:400, Life Technologies, Grand Island, NY, United States). To examine vascular endothelial cells (ECs) and smooth muscle cells (SMCs), sections were first incubated overnight at 4°C with rat anti-mouse CD31 (platelet endothelial cell adhesion molecule, PECAM-1) antibody (1:100, Becton-Dickinson Biosciences, Franklin Lakes, NJ, United States), followed by donkey anti-rat-Alexa594 IgG (1:400, Life Technologies) at RT for 1 h, and subsequently incubated with mouse anti-alpha smooth muscle actin-FITC (1:100, Sigma-Aldrich). Nuclei were stained with Hoechst 33,342 diluted in PBS (1:1,000, Life Technologies) at RT for 5 min.

### Quantification of chronic inflammation and revascularization

To evaluate chronic inflammation within the infarct region, immunofluorescent staining of anti-mouse CD68 was performed on the serial cryosections of flash-frozen mouse hearts. The macrophage infiltration index, indicated by the number of CD68^+^ phagocytic cells per mm^2^, was subsequently estimated by averaging CD68^+^ cell counts in 6-8 randomly selected images of the entire infarct region of each heart at the mid-infarct level. To quantify revascularization in the heart, double immunofluorescent staining with antibodies against a mouse EC marker, CD31, and a mammalian SMC marker, αSMA, was sequentially performed on the serial cryosections of mouse hearts. The capillary density, indicated by the number of CD31^+^ capillary ECs per mm^2^, was subsequently estimated by averaging CD31^+^ cell counts in 6 randomly selected images of the infarct region or peri-infarct area of each heart at the mid-infarct level; the SMC density, indicated by the number of perivascular (i.e., adjacent to CD31^+^ ECs) αSMA + cells per mm^2^, was subsequently computed by averaging αSMA + cell counts in 6 randomly selected images of the infarct region or peri-infarct area of each heart at the mid-infarct level, as described previously (Chen et al., 2013; Okada et al., 2012). All images were analyzed using ImageJ.

### Measurement of cardiac fibrosis

Masson’s trichrome staining kit (IMEB, San Marcos, CA, United States) was used to reveal collagen deposition on heart serial cryosections, following the manufacturer’s instructions. The area of the collagen deposition (indicating cardiac fibrosis) and the area of the entire left ventricular cardiac tissue (including the septal area and excluding void space in the chamber cavity) were measured using a digital image analyzer (ImageJ). Fibrotic area fraction was estimated as the ratio of fibrotic tissue to the entire cross-sectional area of left ventricular tissue and averaged from 6 randomly selected sections at comparable infarct levels per heart.

### Quantitative polymerase chain reaction analysis

Quantitative polymerase chain reaction (qPCR) was performed to quantify relative expression levels of key genes in the infarcted hearts 5 days after the induction of MI. The SYBR Green Master Mix for qPCR (A25741; Applied Biosystems, Waltham, MA, United States) was used with forward and reverse primer pairs (Integrated DNA Technologies, Newark, NJ, United States) for the qPCR gene expression analysis (Table 1). Glyceraldehyde-3-phosphate dehydrogenase (GAPDH) was used as the endogenous control gene for delta Ct calculation. Genes investigated include: TNF-α, IL-6, IFNγ, inducible nitric oxide synthase (iNOS), IL-1β, mIL-10, mannose receptor (CD206), arginase 1 (Arg1), AXL receptor tyrosine kinase (Axl), T-cell membrane protein 4 (Tim4), MER proto-oncogene tyrosine kinase (MerTK), chemokine (C-X3-C motif) ligand 1 (CX3CL1), vascular endothelial growth factor-A (VEGF-A), hyaluronidase 3 (Hyal3), alpha smooth muscle actin (α-SMA), v-set immunoglobulin domain-containing 4 (Vsig4), Fc-gamma receptor 1 (CD64), scavenger receptor cysteine-rich type 1 protein M130 (CD163), platelet-derived growth factor (PDGF), periostin (Postn), and type I collagen (Col1a1) (Jung et al., 2017). The fold change in gene expression was calculated using the 2^(−ΔΔCt)^ method (*n* = 5 per group).

**TABLE 1:** Primer sequences for quantitative real-time PCR (qPCR) analysis.

Gene symbol	Forward primer (5′→ 3′)	Reverse primer (5′→ 3′)
GAPDH	AGG​TCG​GTG​TGA​ACG​GAT​TTG	TGT​AGA​CCA​TGT​AGT​TGA​GGT​CA
IL1-beta	GCA​ACT​GTT​CCT​GAA​CTC​AAC​T	ATC​TTT​TGG​GGT​CCG​TCA​ACT
IFN-gamma	ATG​AAC​GCT​ACA​CAC​TGC​ATC	CCA​TCC​TTT​TGC​CAG​TTC​CTC
IL-6	TAG​TCC​TTC​CTA​CCC​CAA​TTT​CC	TTG​GTC​CTT​AGC​CAC​TCC​TTC
iNOS	CAC​CAA​GCT​GAA​CTT​GAG​CGA	CCA​TAG​GAA​AAG​ACT​GCA​CCG​A
TNF-alpha	CCC​TCA​CAC​TCA​GAT​CAT​CTT​CT	GCT​ACG​ACG​TGG​GCT​ACA​G
Axl	TGA​GCC​AAC​CGT​GGA​AAG​AG	AGG​CCA​CCT​TAT​GCC​GAT​CTA
MerTk	ACC​CAG​TTG​CTA​GAG​AGC​TG	TGG​TGA​GTC​TGT​CTC​CGG​TAA
Vsig4	AGA​GGC​TAC​AGG​CAA​GTT​TTG	GGA​GTC​ACG​TAG​GAA​GAT​GGT
CX3CL1	CGC​GTT​CTT​CCA​TTT​GTG​TA	TGG​GAT​TCG​TGA​GGT​CAT​CT
Arg1	GGA​AAG​CCA​ATG​AAG​AGC​TG	GAT​GCT​TCC​AAC​TGC​CAG​AC
CD206	GGT​CTA​TGG​AAC​CAC​GGA​TG	TGC​CCA​GTA​AGG​AGT​ACA​TGG
mIL-10	CGA​CTC​CTT​AAT​GCA​GGA​CT	TTGATTTCTGGGCCATGC
α-SMA	GTC​CCA​GAC​ATC​AGG​GAG​TAA	TCG​GAT​ACT​TCA​GCG​TCA​GGA
Hyal3	TCT​GTG​GTA​TGG​AAT​GTA​CCC​T	TTT​TGG​CCG​TGA​AAA​TGT​TGG
VEGF	GCA​CAT​AGA​GAG​AAT​GAG​CTT​CC	CTC​CGC​TCT​GAA​CAA​GGC​T
CD163	ATG​GGT​GGA​CAC​AGA​ATG​GTT	CAG​GAG​CGT​TAG​TGA​CAG​CAG
Postn	CCT​GCC​CTT​ATA​TGC​TCT​GCT	AAA​CAT​GGT​CAA​TAG​GCA​TCA​CT
PDGF	AGG​TAT​GTA​TCC​ACA​CAT​GCG​T	AGT​TCC​TGT​TGG​TTT​CAT​CTC​G
Col1a1	GCT​CCT​CTT​AGG​GGC​CAC​T	CCA​CGT​CTC​ACC​ATT​GGG​G
CD64	AGG​TTC​CTC​AAT​GCC​AAG​TGA	GCG​ACC​TCC​GAA​TCT​GAA​GA

### Statistical analysis

All measured data are presented as mean ± standard error (SE). Statistical differences between groups were analyzed by one-way analysis of variance (ANOVA) or two-way repeated ANOVA (for repeated echocardiographic measurements) with a 95% confidence interval. Statistical significance was set at p≦0.05. For multiple comparisons *post hoc* analysis, Bonferroni or Dunnett test was performed for one-way ANOVA, and Tukey test was performed for two-way ANOVA. Statistical analyses were performed with GraphPad Prism 10 statistics software (Dotmatics, Boston, MA, United States).

### Data availability

The authors declare that all relevant data supporting the findings of this study are available within the paper and its supplementary Information. Source data files for the quantitative data generated in this study are available upon request.

## Results

### IL-10 exhibits dose-dependent modulations on polarized macrophage phenotypes

**FIGURE 1:**
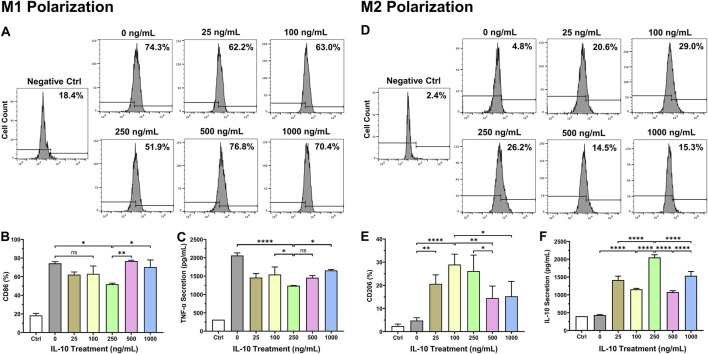
Dose-dependent effects of IL-10 on polarized macrophage phenotypes. To investigate dose-dependent effects of IL-10 on the phenotypes of polarized Mφ, naïve RAW264.7 cells were first differentiated into the classic M1 and M2 phenotypes with mIFNγ and mIL-4, respectively, followed by treatment with titrated IL-10 (0, 25, 100, 250, 500, or 1,000 ng/mL) for 24 h. We then analyzed M1 and M2 phenotypic changes by labeling cells with **(A, B)** anti-CD86 (classic M1 marker) and **(D, E)** anti-CD206 (classic M2 marker) antibodies, respectively, before subjecting cells to flow cytometry. **(A)** Representative graphs with averaged ratios of CD86^+^ cells from each IL-10 treatment group. **(B)** Quantification of the flow cytometry data (*n* = 3 per group). Only the 250 group showed significantly lower CD86 expression than the 0 group (p = 0.0148). The 500 and 1,000 groups had significantly higher CD86 expression than the 250 group (p < 0.01 and p < 0.05, respectively). **(C)** Detection of mTNF-α secretion by polarized M1 cells after hIL-10 treatment using ELISA. The 250 group consistently exhibited significantly lower TNF-α secretion than the 0, 100, and 1,000 groups (p < 0.0001, p = 0.0478, and p = 0.0146, respectively). **(D)** Representative graphs with averaged ratios of CD206+ cells from each IL-10 treatment group. **(E)** Quantification of the flow cytometry data (*n* = 3 per group). The 25, 100, and 250 groups showed significantly higher CD206 expression than the 0 group (p = 0.007, p < 0.0001, and p = 0.0003, respectively). The 500 and 1,000 groups expressed much less CD206 than the 100 (p = 0.0078 and p = 0.0123) and 250 groups (p = 0.0425 and p = 0.0654), respectively. **(F)** Detection of native mIL-10 secretion by polarized M2 cells after hIL-10 treatment using ELISA. All IL-10 treatment groups exhibited significantly higher mIL-10 secretion than the 0 group (all p < 0.0001). Wildtype naïve RAW264.7 cells served as the negative controls (Ctrl). Note: *p < 0.05, **p < 0.01, ***p < 0.001, and ****p < 0.0001; ns: not statistically significant.

To assess the efficacy of IL-10 for modulating the phenotypes of polarized Mφ, we first differentiated naïve RAW264.7 monocytes to classic M1 and M2 Mφ with mIFNγ and mIL-4, respectively (Supplemental Figure 1). We then treated polarized Mφ individually with differential doses of IL-10 (0, 25, 100, 250, 500, and 1,000 ng/mL) for 24 h. Flow cytometry analysis showed that the 250 group has significantly lower CD86 (classic M1 marker) expression when compared with no IL-10 treatment (0 ng/mL); the 500 and 1,000 groups have notably higher CD86 expression when compared with the 250 group (Figures 1A, B). The ELISA assay revealed that M1 Mφ in the 250 group secreted notably less pro-inflammatory cytokine murine TNF-α (mTNF-α) when compared with 0, 100, and 1,000 groups (Figure 1C). On the other hand, the 25, 100, and 250 groups had the highest CD206 (classic M2 marker) expression when compared with no IL-10 treatment while the 500 and 1,000 groups had ∼45–50% and ∼40–45% lower CD206 expression than the 100 and 250 groups, respectively (Figures 1D, E). The ELISA assay showed that all IL-10 treated groups have substantially increased secretion of native murine IL-10 (mIL-10), with the 250 group showing the highest mIL-10 secretion (Figure 1F), consistent with our flow cytometry data. No antibody cross-reaction between mIL-10 and exogenous hIL-10 was observed (Supplemental Figure 2). These results suggest that one can induce drifting of established Mφ phenotypes from the pro-inflammatory/destructive toward the anti-inflammatory/resolutive by administering appropriate doses of IL-10 (Yunna et al., 2020).

### IL-10 programs macrophage polarization in a dose-dependent manner

**FIGURE 2:**
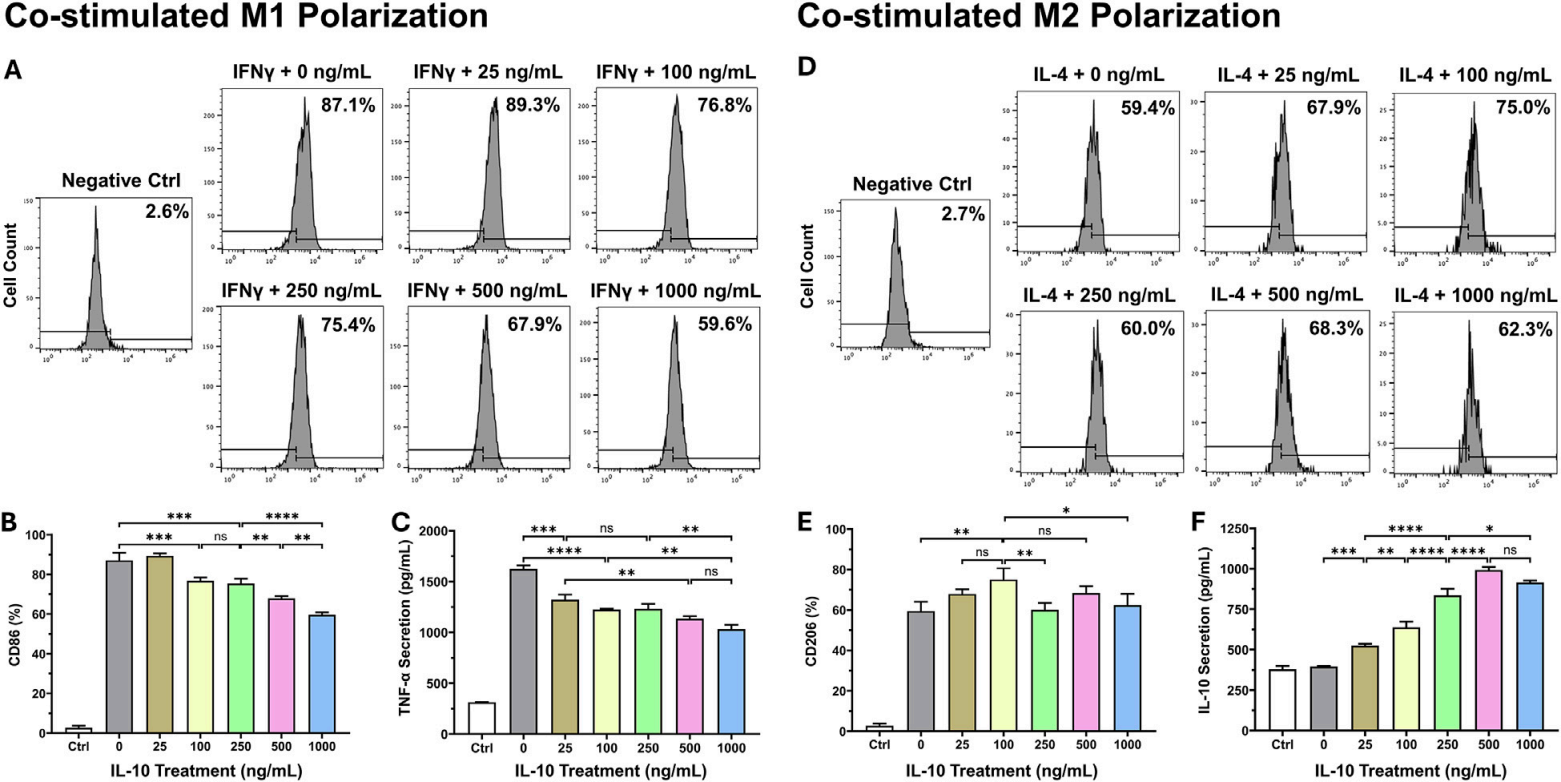
Dose-dependent effects of IL-10 on macrophage polarization. To study the effect of exogenous IL-10 treatment with differential doses on the Mφ polarization process, we differentiated naïve RAW264.7 monocytes with a standard polarizing agent (mIFNγ for M1 and mIL-4 for M2) and one of the six doses of IL-10: 0, 25, 100, 250, 500, and 1,000 ng/mL simultaneously for 36 h. We analyzed the manifestation of classic M1 and M2 phenotypes with flow cytometry by labeling cells with **(A, B)** anti-CD86 and **(D, E)** anti-CD206 antibodies, respectively. **(A)** Representative graphs with averaged ratios of CD86^+^ cells from each IL-10 treatment group. **(B)** Quantification of the flow cytometry data (*n* = 3 per group). All groups had significantly lower CD86 expression than the 0 group (all p < 0.001), except the 25 group (p > 0.05). **(C)** Detection of mTNF-α secretion by polarized M1 cells after hIL-10 treatment using ELISA. All groups had significantly less TNF-α secretion when compared with the 0 group (all p < 0.001). **(D)** Representative graphs with averaged ratios of CD206+ cells from each IL-10 treatment group. **(E)** Quantification of the flow cytometry data (*n* = 3 per group). The 100 group showed significantly higher CD206 expression than the 0, 250, and 1,000 groups (p = 0.0047, p = 0.0065, and p = 0.0229, respectively). **(F)** Detection of native mIL-10 secretion by polarized M2 cells after hIL-10 treatment using ELISA. All groups had significantly higher mIL-10 secretion than the 0 group (all p < 0.001). Wildtype naïve RAW264.7 cells served as the negative controls (Ctrl). Note: *p < 0.05, **p < 0.01, ***p < 0.001, and ****p < 0.0001; ns: not statistically significant.

To investigate how different exogenous IL-10 doses would impact the Mφ polarization process, we co-stimulated naïve RAW264.7 monocyte differentiation with one of the six doses of IL-10: 0, 25, 100, 250, 500, and 1,000 ng/mL and a standard polarizing agent (mIFNγ for M1 and mIL-4 for M2). For M1 polarization, flow cytometry analysis showed that all groups treated with ≥100 ng/mL IL-10 have significantly decreased M1 polarization, but not the 25 ng/mL, when compared with the 0 ng/mL (Figures 2A, B). The ELISA assay indicated that all IL-10 treated groups have notably less mTNF-α secretion when compared with the 0 group (Figure 2C). It appeared that higher doses of IL-10 may have stronger effects in reducing M1 polarization. For M2 polarization, the 100 group exhibited the highest CD206 expression when compared with the 0, 250, and 1,000 groups (Figures 2D, E). The ELISA assay revealed that all IL-10 treated groups secrete substantially more native mIL-10 when compared with the 0 group, with the 500 group having the highest secretion (Figure 2F). These data suggested that when co-administered with a polarizing agent, IL-10 has notable dose-dependent effects over Mφ polarization, with the potential to achieve a distinct balance of M1 or M2 phenotype with the addition of an appropriate dose of IL-10.

### Intramyocardial IL-10 administration exhibits dose-dependent effects on cardiac function

**FIGURE 3:**
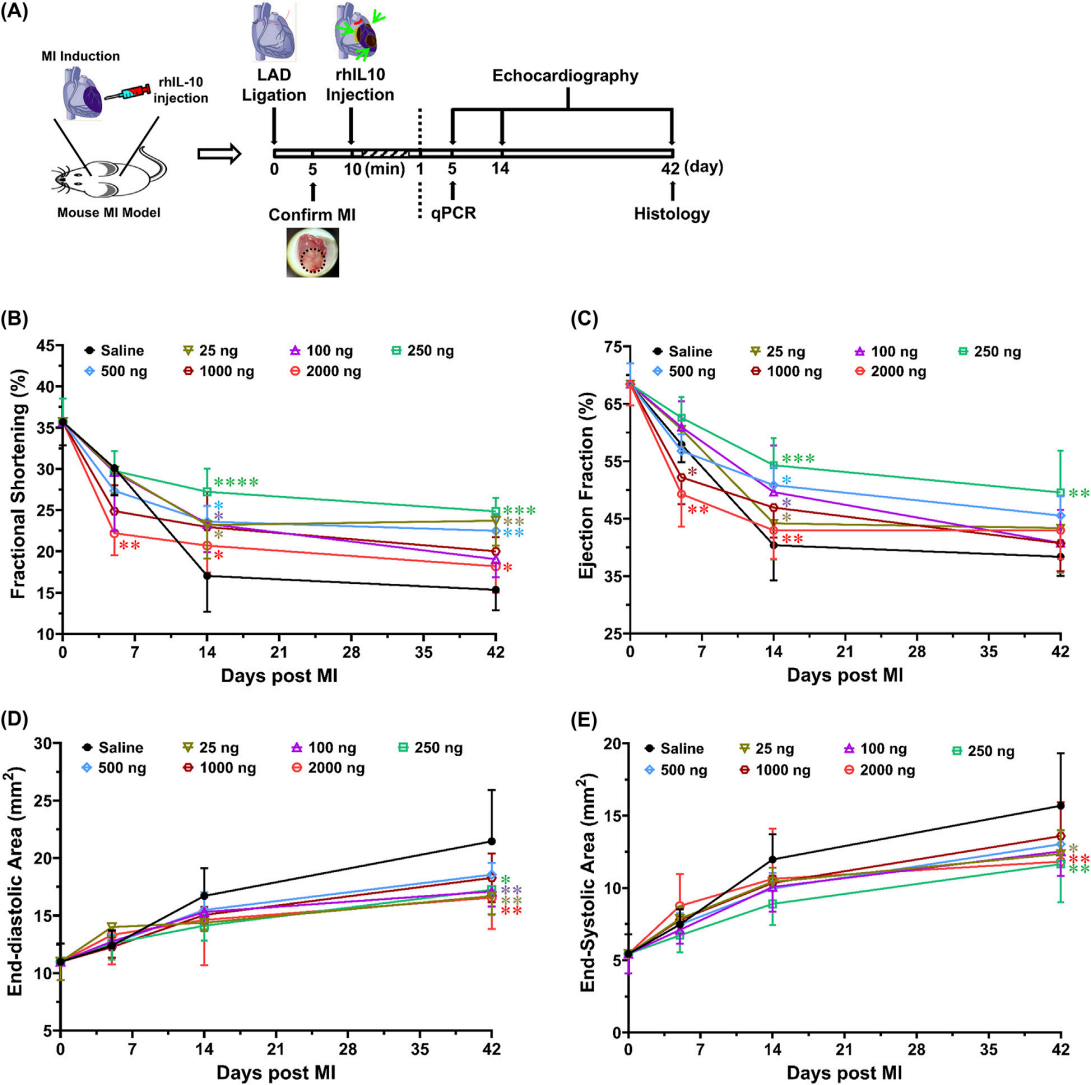
Intramyocardial injection of IL-10 improves cardiac function with dose-dependent effects. Using a mouse MI model, we investigated whether intramyocardial IL-10 administration exerts any dose-dependent efficacy or potential unwanted effect on cardiac function post-MI. **(A)** The timeline of the animal study, including surgical procedures, cardiac functional evaluations, and tissue-based analyses. Ten minutes after the induction of MI, mice received one-time intramyocardial injection of IL-10 with one of the six doses (ng): 25 (sage), 100 (purple), 250 (green), 500 (blue), 1,000 (brown), or 2000 (red) or 0.9% saline (vehicle control, black). Echocardiography was repeatedly performed by a blinded investigator to measure left ventricular **(B)** fractional shortening (5D: **p < 0.01, 2000 vs saline, 25, 100, and 250; 14D: *p < 0.05, 2000 vs 250; 42D: *p < 0.05, 2000 vs 250; all other comparisons vs saline), **(C)** ejection fraction (5D: **p < 0.01, 2000 vs 25, 100, and 250; *p < 0.05, 1,000 vs 250; 14D: **p < 0.01, 2000 vs 250; *p < 0.05, 25 vs 250; all other comparisons vs saline), **(D)** end-diastolic area (all comparisons vs saline), and **(E)** end-systolic area (all comparisons vs saline) at 5, 14, and 42 days post-surgery (*n* = 5 per group). Results were statistically analyzed with two-way repeated ANOVA with Tukey multiple comparison test. Notably, the 250 group exhibited the highest overall cardiac functional benefits among all treatment doses. The 1,000 and 2000 groups had decreases in cardiac contractile function at 5 days post-MI, suggesting possible transient side-effects caused by high-dose IL-10 treatment. Note: *p < 0.05, **p < 0.01, ***p < 0.001, and ****p < 0.0001.

To evaluate the potential of IL-10 as an immunomodulatory treatment for enhancing cardiac repair post-MI, we examined the cardiac function after single intramyocardial administration of different IL-10 doses in a mouse MI model (Figure 3A). A dose range of 25–2000 ng IL-10 was chosen for testing based on the outcomes of the *in vitro* studies. The surgical mortality rate (death during the operation or within 60 min after the surgery) was ∼17% (1 in 7) in all groups. Among all animals that fully recovered from the surgery, one died before the terminal time point in each of these four groups: saline control, 25, 100, and 2000, and was excluded from this study. In total, five animals (*n* = 5) per group with no notable behavioral abnormality were included in the 6-week repeated echocardiographic assessment.

Cardiac contractile function was estimated by LV fraction shortening (LVFS, Figure 3B) and LV ejection fraction (LVEF, Figure 3C), whereas LV chamber size was determined by LV end-diastolic area (LVEDA, Figure 3D) and end-systolic area (LVESA, Figure 3E). At 5 days post-MI, the 2000 group had significantly lower LVFS than all groups, except the 500 and 1,000 groups (Supplemental Figure 3). Particularly, the 1,000 and 2000 groups exhibited notably lower LVEF than the 250 group (Figure 3C). At 6 weeks post-MI, the 25, 250, and 500 groups showed considerably higher LVFS than the saline control (Figure 3B); however, only the 250 group exhibited notably higher LVEF than the saline control (Figure 3C, p = 0.0067) while showing significantly higher LVFS than the 2000 group (Figure 3B; p = 0.0202). These results suggest that single intramyocardial treatment with ≥1,000 ng IL-10 has an early negative impact in LV contractile function while injecting 250 ng IL-10 appears to offer more consistent LV contractile protection than other doses in the long term.

Moreover, at 6 weeks post-MI, the 25, 250, and 2000 groups exhibited significantly smaller LVEDA (Figure 3D) and LVESA (Figure 3E) when compared with the saline control. The 100 group notably reduced LVEDA (p = 0.007) but not LVESA (Figures 3D, E). These data suggest that single intramyocardial injection with select IL-10 doses can help ameliorate LV dilatation. Overall, our echocardiographic analyses suggest that 1) 250 ng IL-10 treatment resulted in the most significant long-term functional benefits among all doses, and 2) IL-10 has noteworthy dose-dependent effects when administered intramyocardially, including early adverse effects in LV contractile function with excessive exogenous IL-10.

### Intramyocardial IL-10 treatment modulates early myocardial recovery post-MI

**FIGURE 4:**
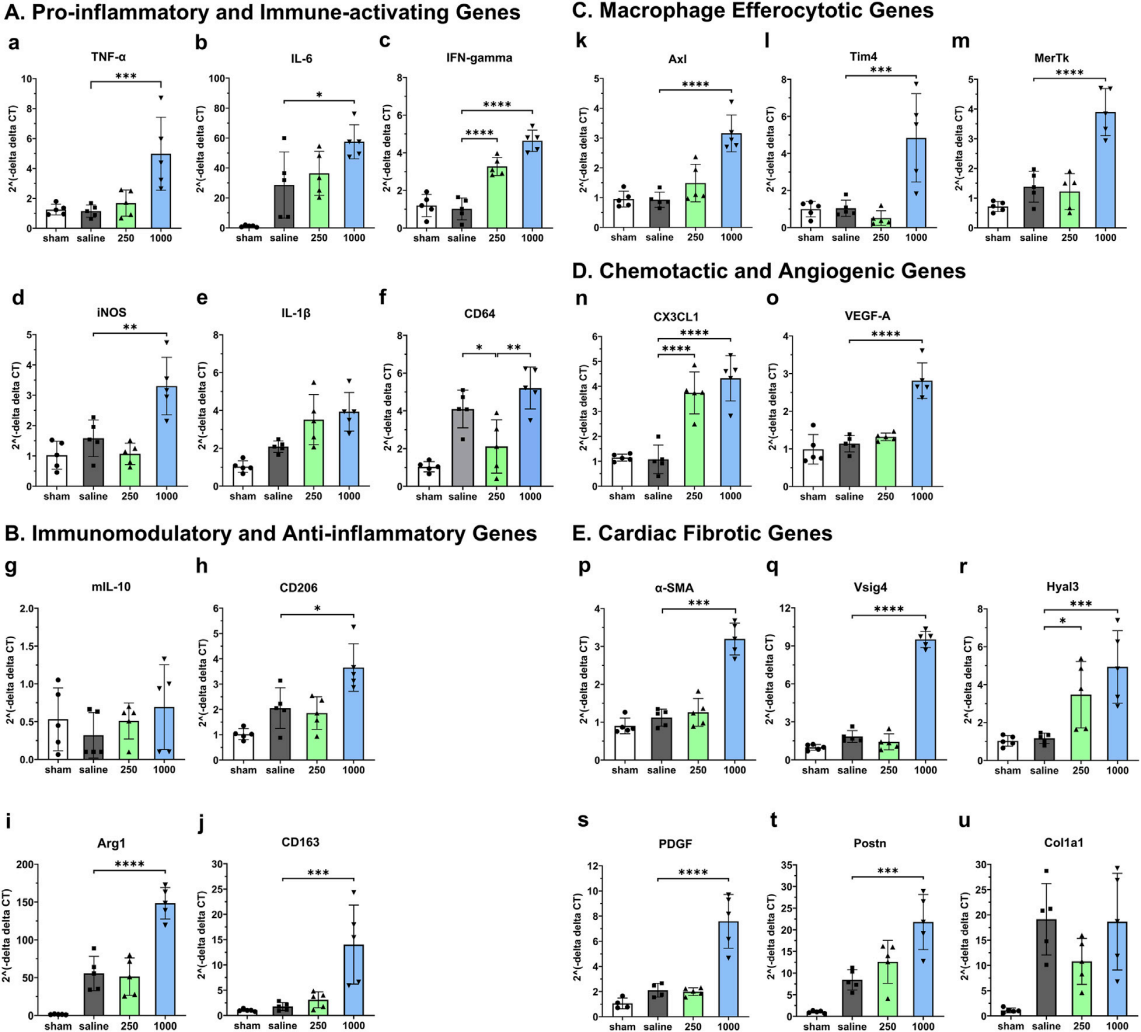
Early dose-dependent effects of intramyocardial IL-10 treatment on myocardial gene expression. We used qPCR to analyze the expression levels of five groups of genes in mouse LV treated with different doses of IL-10 (0, 250, or 1,000 ng) at 5 days of post-MI, including **(A)** pro-inflammatory and immune-activating genes (TNF-α, IL-6, IFNγ, iNOS, IL-1β, and CD64); **(B)** immunomodulatory and anti-inflammatory genes (mIL-10, CD206, Arg1, and CD163); **(C)** macrophage efferocytotic genes (Axl, Tim4, and MerTK); **(D)** chemotactic and angiogenic genes (CX3CL1 and VEGF-A); **(E)** cardiac fibrotic genes (α-SMA, Vsig4, Hyal3, PDGF, Postn, and Col1a1). Data were statistically compared using one-way ANOVA with Dunnett multiple comparisons test (*n* = 5 per group). The results suggest that 250 and 1,000 ng IL-10 treatment differentially activated diverse functional signaling networks, with 1,000 ng IL-10 seemingly triggering both pro-inflammatory and anti-inflammatory pathways. Note: *p < 0.05, **p < 0.01, ***p < 0.001, and ****p < 0.0001.

To assess the early impact of intramyocardial IL-10 administration in modulating myocardial inflammation, Mφ function, and cardiac remodeling, we investigated the expression of 16 essential genes involved in relevant functional pathways in the infracted mouse hearts treated with 0 (saline control), 250, and 1,000 ng IL-10 at 5 days post-MI. Our qPCR analysis revealed that most of these genes exhibit distinct expression patterns after treating with 250 and 1,000 ng IL-10 (Figure 4).

Comparing with the saline control, despite inducing notable upregulation in multiple beneficial genes, including the anti-inflammatory CD206 (Figures 4B–H, p = 0.0166) and Arg1 (Figures 4B–I, p < 0.0001), the Mφ efferocytotic Tim4 (Figures 4C–L, p = 0.0005) and MerTK (Figures 4C–M, p < 0.0001), the chemotactic CX3CL1 (Figures 4D–N, p < 0.0001), the angiogenic VEGF-A (Figures 4D–O, p < 0.0001), and the anti-fibrotic Vsig4 (Figures 4E–Q, p < 0.0001) genes, the 1,000 group also considerably upregulated pro-inflammatory and pro-fibrotic genes, including TNF-α (Figure 4A, p = 0.0009), IL-6 (Figures 4A, B, p = 0.0159), iNOS (Figures 4A–D, p = 0.0015), Axl (Figures 4C–K, p < 0.0001), α-SMA (Figures 4E–P, p < 0.001), the hyaluronan-degrading Hyal3 (Figures 4E–R, p = 0.0009), PDGF (Figures 4E–S, p < 0.0001), Postn (Figures 4E–T, p = 0.0004), and Col1a1 (Figures 4E–U, p = 0.9989).

In contrast, comparing with the saline control, the 250 group showed notable upregulation of the immune-activating IFNγ (Figures 4A–C, p < 0.0001), CX3CL1 (Figures 4D–N, p < 0.0001), and Hyal3 (Figures 4E–R, p = 0.0348) genes significant downregulation of macrophage M1 phenotype marker CD64 (Figures 4A–F, p = 0.021) as well as modest downregulation of iNOS and Col1a1 genes. These results suggest that distinct IL-10 doses differentially regulate essential genes involved in the pro- and anti-repair pathways, potentially contributing to the sustained long-term LV function in the 250 group as well as the short-term contractile dysfunction observed in high-dose IL-10 treatment at 5 days post-MI.

### Single administration of IL-10 exhibits dose-dependent reduction of chronic inflammation

**FIGURE 5:**
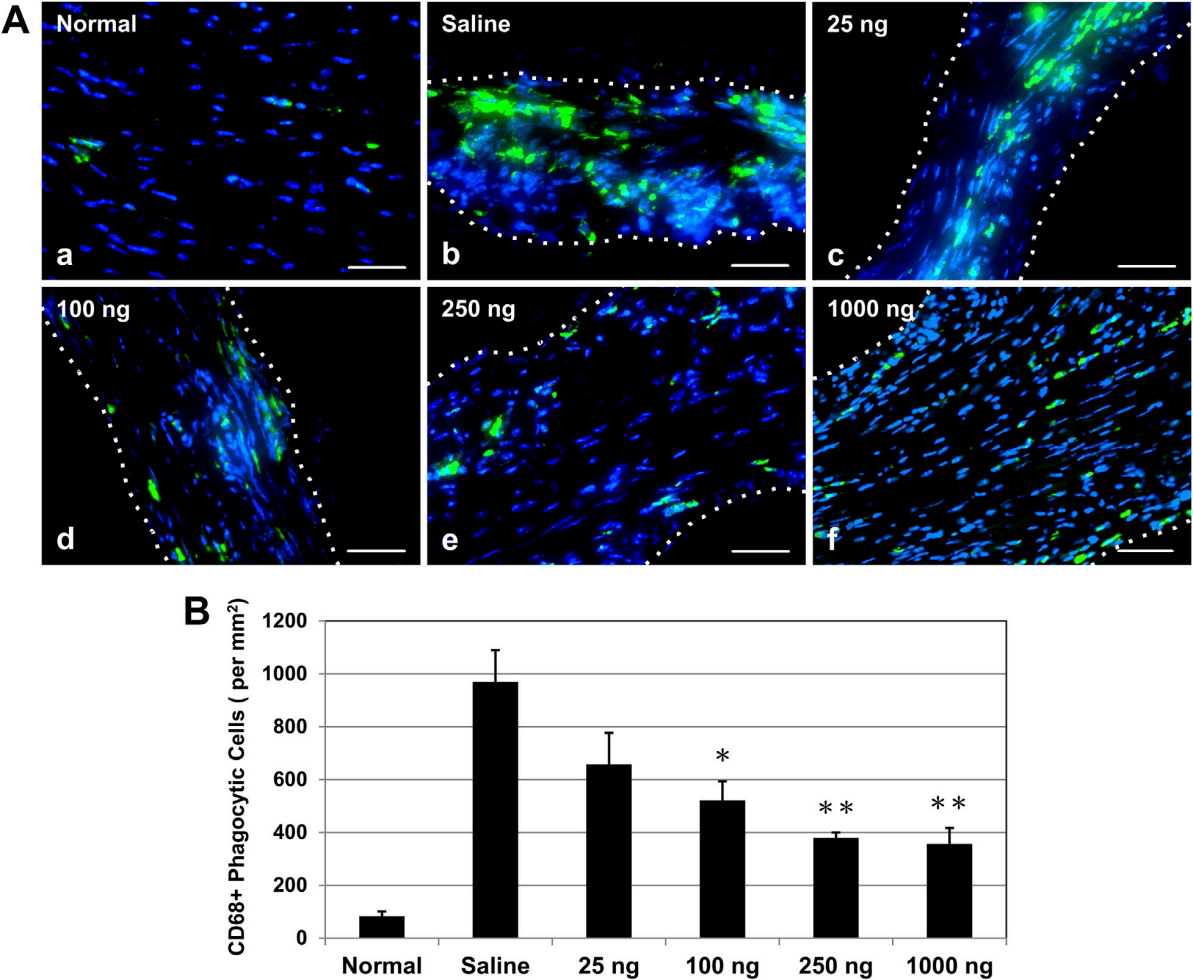
Intramyocardial IL-10 treatment decreases chronic inflammation within the infarct region. Chronic inflammation was assessed by the number of infiltrating CD68^+^ phagocytic cells (green) within the infarct region at 6 weeks post-infarction. **(A)** Representative images of anti-CD68 immunofluorescent staining revealing phagocytic cell infiltration within the infarct region at the mid-infarct level from each group (scale bars: 50 μm). Nuclei were stained in blue. Healthy (normal) hearts served as negative controls. **(B)** Three IL-10 doses (100, 250, and 1,000 ng) resulted in significant decreases of CD68^+^ cell infiltration (*n* = 3 per group; p = 0.046, p = 0.007, and p = 0.006, respectively, vs saline). Note: *p < 0.05, **p < 0.01.

Based on the results of the cardiac functional studies above, we selected a dose range of 25–1,000 ng IL-10 per heart to investigate whether single intramyocardial IL-10 treatment has any dose-dependent immunomodulatory effect on chronic inflammation. At 6 weeks post-MI, phagocytic cells at the infarct site were detected with anti-CD68 immunofluorescent staining (Figure 5A). Unaffected myocardium contained few CD68^+^ cells (Figures 5A, A). All IL-10-treated groups exhibited a trend of diminished numbers of CD68^+^ cells within the infarct region (Figures 5A, C–F) compared with the saline-injected control (Figures 5A, B). Quantitatively, three IL-10-treated groups showed significantly less CD68^+^ cell infiltration than the saline control group (969.81 ± 120.02/mm^2^): 100 (522.20 ± 71.62/mm^2^), 250 (379.41 ± 20.50/mm^2^), and 1,000 (356.83 ± 60.17/mm^2^) (Figure 5B, *n* = 3 per group). These results suggest that single intramyocardial administration with ≥100 ng IL-10 soon after MI has a long-lasting modulatory effect on MI-induced chronic inflammation.

### Intramyocardial IL-10 treatment ameliorates left ventricular fibrosis

**FIGURE 6:**
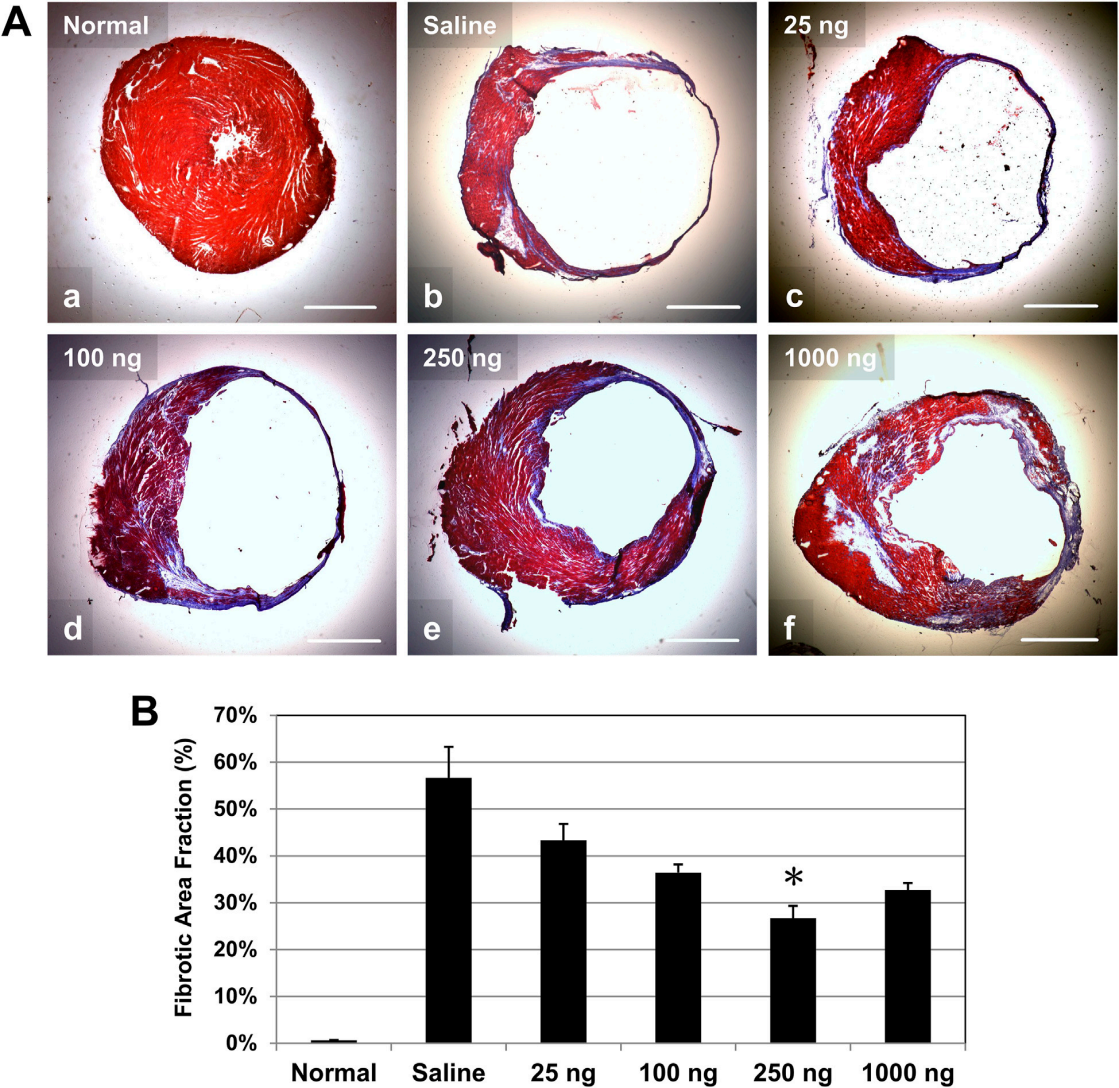
Intramyocardial IL-10 treatment reduces left ventricular myocardial fibrosis. Long-term left ventricular myocardial fibrosis was revealed by Masson’s trichrome histological staining at 6 weeks post-infarction. **(A)** Representative images of Masson’s trichrome-stained transverse sections of hearts at the mid-infarct level (collagen deposition stained in blue/purple, cardiac muscle stained in red; scale bars: 1 mm). Healthy (normal) hearts served as negative controls. **(B)** Quantitatively, only the 250 group exhibited significantly reduced LV fibrotic area fraction (*n* = 3 per group; *p = 0.05, vs saline).

To investigate the effect of early intramyocardial IL-10 treatment on cardiac fibrosis, we evaluated LV scar tissue formation (stained in blue/purple) with Masson’s trichrome histological staining. Healthy hearts had minimal deposition of collagen (Figures 6A, A). At 6 weeks post-infarction, hearts treated with different doses of IL-10 (Figures 6A, C–F) exhibited a trend of smaller infarct regions and less collagen deposition than the saline-injected control at the mid-infarct level (Figures 6A, B). Quantitative estimation of the LV fibrotic area fraction revealed a common trend of reduction in fibrosis in all IL-10 treated groups compared with the saline control group (Figure 6B, *n* = 3 per group). Only the 250 group exhibited a statistically significant 53% reduction in LV fibrosis (Figure 6B), suggesting that single intramyocardial IL-10 administration has a dose-dependent, long-term anti-fibrotic effect.

### Intramyocardial administration of IL-10 enhances revascularization

**FIGURE 7:**
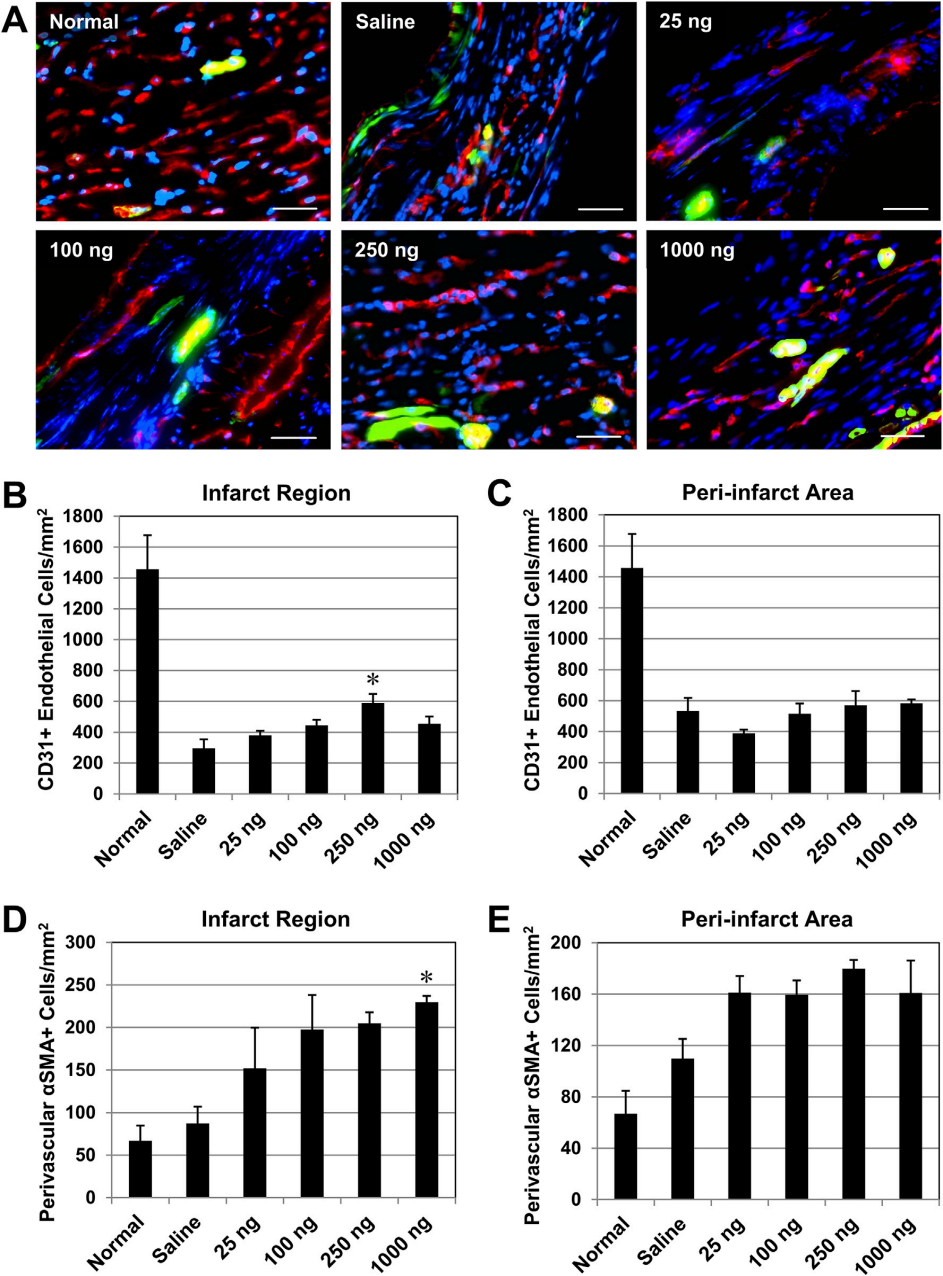
Intramyocardial injection of IL-10 enhances revascularization in the ischemic myocardium. Long-term revascularization, including angiogenesis and vasculogenesis, in the ischemic myocardium was evaluated by the presence of CD31^+^ endothelial cells (ECs) and αSMA + peri-vascular smooth muscle cells (SMCs), respectively, at 6 weeks post-infarction (*n* = 3 per group). Perivascular SMCs defined as αSMA + cells around CD31^+^ ECs. Healthy (normal) hearts served as baseline controls (*n* = 3). **(A)** Representative images of CD31^+^ ECs (red) and αSMA + SMCs (green) within the infarct region at the mid-infarct level from each group (scale bars: 50 μm). Nuclei were stained in blue. **(B)** Quantitatively, only the 250 ng group had a significantly higher density of CD31^+^ ECs at the infarct (p = 0.013, vs saline). **(C)** No significant difference in the CD31^+^ EC density was noted in the peri-infarct area (all p > 0.05, vs saline). **(D)** Only the 1,000 group had a significantly higher density of αSMA + SMCs within the infarct region (p = 0.045, vs saline). **(E)** No notable difference in the αSMA + SMC density in the peri-infarct area (all p > 0.05, vs saline). Note: *p < 0.05.

We examined whether single administration of IL-10 enhances long-term revascularization within the ischemic myocardium. At 6 weeks post-infarction, the presence of CD31^+^ endothelial cells (ECs) and αSMA + perivascular cells (i.e., pericytes and vascular smooth muscle cells adjacent to CD31^+^ ECs) at the infarct region were simultaneously detected with dual immunofluorescent staining (Figure 7A). Quantitative analysis (*n* = 3 per group) showed that only the 250 group had a significantly higher density of CD31^+^ ECs at the infarct region than the saline control (Figure 7B); all other IL-10 treatment groups had marginal benefits (Figure 7B). No IL-10 treatment groups exhibited a notable increase in CD31^+^ EC density in the peri-infarct areas compared with the saline control group (Figure 7C).

On the other hand, only the 1,000 group had a significantly higher density of αSMA + perivascular cells at the infarct region than the saline control group (Figure 7D); all other IL-10 treatment groups contributed to the repopulation of perivascular cells post-MI marginally (Figure 7D). None of the IL-10 treatment groups showed significant increases in αSMA + cell density in the peri-infarct areas compared with the saline control group (Figure 7E). These data suggest that when intramyocardial administered, select doses of IL-10 have notable effects in enhancing long-term repopulation of myocardial microvasculature at the infarct region, but not in the peri-infarct areas.

## Discussion

**FIGURE 8:**
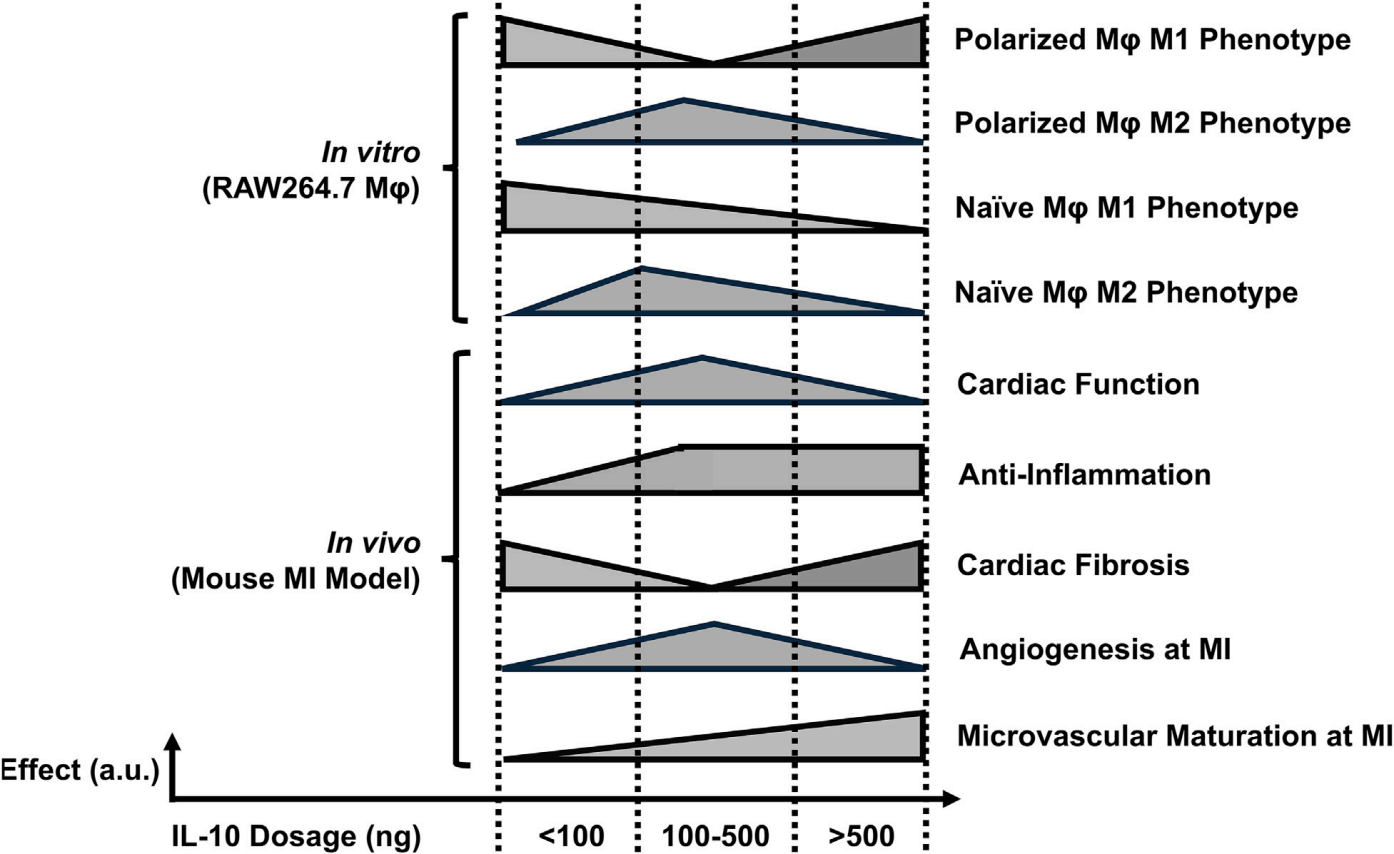
The estimated dose-dependent effects of single IL-10 administration. The illustration summarizes the overall dose-effect relationships after single IL-10 administration *in vitro* and *in vivo*. The x- and y-axis of each horizontal bar represent the IL-10 dose ranges and the estimated levels of individual annotated effects, respectively. Note: a. u.: arbitrary unit.

With its pleiotropy in the immune system, IL-10 and its family of cytokines have been widely explored as therapeutic approaches in a wide variety of human diseases involving autoimmunity or excessive inflammation, such as rheumatoid arthritis, psoriasis, and inflammatory bowel disease (Wang et al., 2019; Kwilasz et al., 2015; Tilg et al., 2002; Huynh et al., 2023). Recent reports suggest that high-dose IL-10 treatment may not lead to the expected therapeutic outcome, raising the question of whether IL-10 has dose-dependent effects for enhancing cardiac tissue repair after MI, or even unexpected side effects from overdosing. By identifying an optimal range of IL-10 doses for cardiac repair via intramyocardial administration, we believe it is possible to safely utilize IL-10 to facilitate myocardial recovery post-MI, prevent adverse effects from IL-10 overdose, and lower the total cost of the costly recombinant protein treatment. Our findings shed light on the immunomodulatory properties of IL-10 on programming Mφ phenotypes and its potential therapeutic implications for myocardial repair post-MI (Figure 8).

We examined the impact of differential doses of IL-10 on murine Mφ polarization with *in vitro* models. We demonstrated that IL-10 has notable dose-dependent effects not only on the phenotypes of differentiated M1 and M2 Mφ but also on prospective M1 and M2 polarization of naïve Mφ. Our data suggest that increasing concentrations of IL-10, up to 250 ng/mL, reduced the ratio and pro-inflammatory phenotype of M1 Mφ and increased the ratio and anti-inflammatory phenotype of M2 Mφ, with 250 ng/mL IL-10 having the highest overall potency in both populations (Figure 1). However, IL-10 concentrations ≥500 ng/mL did not efficiently reduce M1 or increase M2 ratio and phenotype, suggesting a limitation in the effective dose range of IL-10 for modulating Mφ phenotypes and possibly conflicting actions on differentiated Mφ (Figure 1). On the other hand, when co-administered with mIFNγ or mIL-4 to stimulate M1 or M2 polarization of naïve Mφ respectively, IL-10 ≥ 100 ng/mL significantly reduced M1 polarization while all tested IL-10 doses appeared to facilitate M2 polarization (Figure 2). These results suggest that given an appropriate dose, IL-10 can modulate the balance of Mφ ratio, phenotypes, and/or polarization toward an anti-inflammatory M2 state that facilitates resolution of inflammation and tissue repair (Figure 8).

Based on our *in vitro* findings, we subsequently assessed the translational potential of intramyocardial IL-10 administration and explicitly tested the impact of IL-10 dose-dependency in cardiac repair by injecting a wide range of rhIL-10 doses (up to 80-fold differences) (Figure 3). Despite the very short half-life of IL-10 *in vivo* (2.7–4.5 h), (Huhn et al., 1997), our data showed that an early intramyocardial injection of IL-10 resulted in long-term, dose-dependent effects of on cardiac function and remodeling. The 250 ng IL-10 group had the most significant cardiac contractile benefits among all tested doses, sustaining LV functions at roughly 60%–70% of their normal values. Nonetheless, treatment with ≥1,000 ng IL-10 showed short-term negative impact in cardiac function at 5 days post-MI, indicating a potential toxicity associated with injecting a high dose of IL-10 intramyocardially (Figure 3). Moreover, multiple groups exhibited improved EDA and ESA at 6 weeks post-MI, suggesting long-term merits on cardiac remodeling. These results further suggest that if IL-10 would be used therapeutically for MI, it may be necessary to identify an optimal IL-10 dose range for individual patients, based on their disease timeline or severity, to achieve the maximal efficacy and minimize the potential risk of IL-10 overdose.

Our qPCR analysis highlighted the early changes of gene expression associated with IL-10 administration in the infarcted myocardium. The results suggested that different doses of exogenous IL-10 differentially modulated the expression of many key genes involved in the tissue inflammation, damage and recovery processes, particularly the substantial upregulation of pro-inflammatory, efferocytotic, and fibrotic genes by 1,000 ng IL-10 treatment (Figure 4). Particularly, the 1,000 group also upregulated Axl expression, which is involved in Mφ efferocytosis and can further increase intramyocardial inflammation (Figures 4C–I) (DeBerge et al., 2021) Besides, our histological assays showed that the 250 and 1,000 groups differentially exhibited reduction of chronic inflammation at the infarct, ameliorated LV fibrosis, and, to a minor extent, enhanced revascularization, all of which potentially contributed to the improved LV contractile function and remodeling (Figures 5–7). Intriguingly, the injection of 1,000 ng IL-10 led to simultaneous upregulations of both pro- and anti-inflammatory responses post-MI. Thus, we further hypothesize that the dual activation of pro- and anti-resolution functional pathways by 1,000 ng IL-10 yields conflicting signals to immune cells that potentially contribute to the early transient LV contractile dysfunction observed in the echocardiographic analyses. Overall, these data align with our *in vivo* findings and suggest a complex interplay between IL-10 dosage and endogenous signaling networks that warrants future investigations with advanced approaches (Figure 8).

Previous reports demonstrated that mammalian cardiomyocytes (CMs) have active turnover during physiological conditions and may proliferate extensively after MI (Bergmann et al., 2009; Senyo et al., 2013). We further hypothesized that the reduction of chronic inflammation by intramyocardial IL-10 administration promotes CM survival and/or proliferation following MI. We then investigated whether there also exists any dose-dependent effect of IL-10 in enhancing long-term CM survival or proliferation post-MI (Supplemental Figure 4). Residual CMs at the infarct and in peri-infarct areas were identified by a mature CM marker cTn-I; proliferating CMs were detected by dual-positive signals of cTn-I and a cell proliferation marker Ki-67. To our surprise, we did not observe any notable differences in the number of either residual CMs (Supplemental Figure 4A, all p > 0.05) or proliferating CMs (Supplemental Figure 4B, all p > 0.05) within both the infarct region and peri-infarct areas among all tested IL-10 dose groups (25, 100, 250, and 1,000 ng IL-10; *n* = 3 per group). These results suggest that a single intramyocardial injection of IL-10 did not elicit notable dose-dependent efficacy in promoting long-term CM survival or proliferation, similar to a saline injection in our previous report (Chen et al., 2016).

One reason that large doses of IL-10 were used in previous investigations lies in its short half-life in the body, limiting the bioavailability in the target organ, particularly when administered via systemic routes. To reduce the amount of IL-10 required for effective treatment, appropriate delivery systems, such as exosomes or nanoparticles, may be needed for efficient, targeted delivery of IL-10 (Bu et al., 2021; Duncan et al., 2019). We and others have previously demonstrated that when locally delivered in a controlled manner, IL-10 can serve as an adjuvant for angiogenic or morphogenic cytokines to synergistically preserve heart function and improve cardiac repair after MI (Chen et al., 2015; Johnson et al., 2015). Nonetheless, whether controlled delivery of IL-10 reduces the required therapeutic dose for desirable outcomes and prevents early cardiac dysfunction observed in this study remains to be further investigated.

In addition, our findings that the beneficial effects of IL-10 can be precisely regulated by differential doses of exogenous IL-10 underscore the need for integrative systems approaches that are more efficient to interrogate experimental therapeutics, based on IL-10 or other cytokines, than conventional analytics. Due to the inherent limitations in our experimental approach, we were unable to further dissect the IL-10 dose-response relationship. Modern high-throughput computational approaches, such as machine learning (ML), may help us analyze the complex dynamics of cytokine-based therapies beyond the capacities of conventional experimental approaches. With the ability to handle big, complex datasets and precisely discern nuanced patterns in dose-response relationships, ML algorithms may further identify potential dosing ranges for optimal therapeutic efficacy while minimizing potential adverse effects, paving the way for safer and more effective therapeutic strategies as we move towards precision therapy for MI.

## Conclusion

The findings from this study suggest that given appropriate doses, IL-10 has the potential to modulate Mφ phenotypes *in vitro* and to ameliorate cardiac functional deterioration, reduce myocardial inflammation and fibrosis, and promote revascularization in the context of MI *in vivo* (Figure 8). Our study highlights the dose-dependent effects of IL-10 and underscores the potential clinical significance of appropriate IL-10 administration in managing MI. Overall, these results provide valuable insights into the development of new therapeutic strategies or adjunct therapies targeting specific myocardial pathologies to regulate Mφ phenotypes and augment cardiac repair post-MI.

## Data Availability

The original contributions presented in the study are included in the article/Supplementary Material, further inquiries can be directed to the corresponding author.
