# Supplementary material for: Interleukin-10 exhibit dose-dependent effects on macrophage phenotypes and cardiac remodeling after myocardial infarction
Source: Front Physiol. 2025 Jan 15;15:1481460. doi: 10.3389/fphys.2024.1481460 (PMC11774956; doi:10.3389/fphys.2024.1481460)
Supplement: Supplementary file 1 [file DataSheet1.pdf]

Supplemental Figures and Supplemental Figure Legends

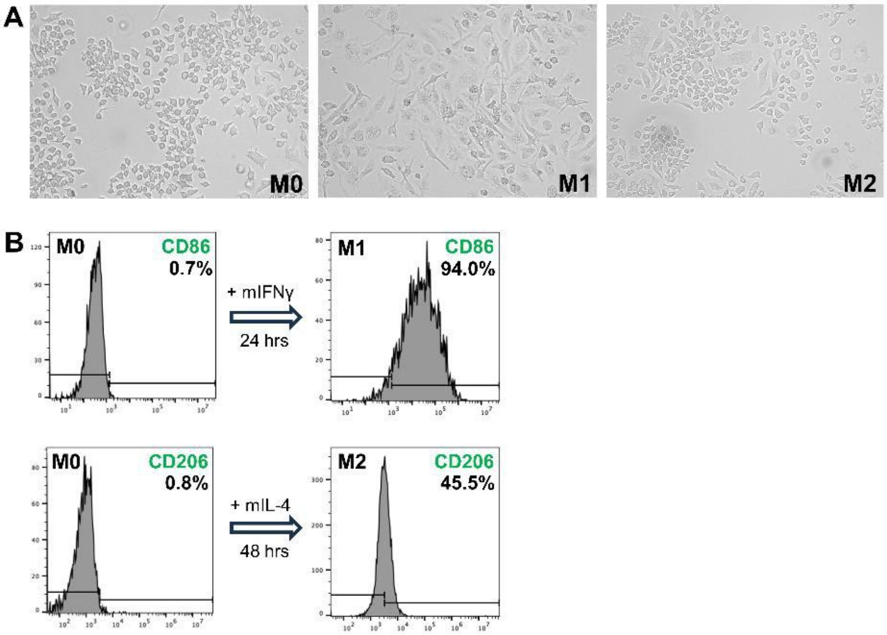

Supplemental Figure 1. Polarizing RAW264.7 cells into classic M1 and M2 phenotypes. (A) Representative images of M0 naïve, M1 polarized, and M2 polarized RAW264.7 cell morphology. **(B)** Validation of RAW264.7 cell polarization with antibody-based detection of classic M1 (CD86) or M2 (CD206) markers, using flow cytometry. Representative graphs from flow cytometry analysis and averaged ratios of marker positive cells are shown (*n*=3 per group).

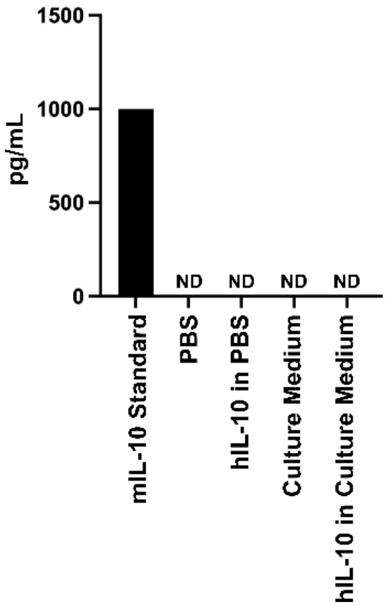

Supplemental Figure 2. Examining the antibody cross-reaction between mIL-10 and hIL-10 in ELISA. We tested whether the ELISA kit used for detecting native mIL-10 secretion cross-reacts with exogenous hIL-10. The results showed positive detection of mIL-10 (1000 pg/mL) with no detection of rhIL-10 (1000 pg/mL) in either PBS or culture medium (*n*=3 per group; ND: not detected).

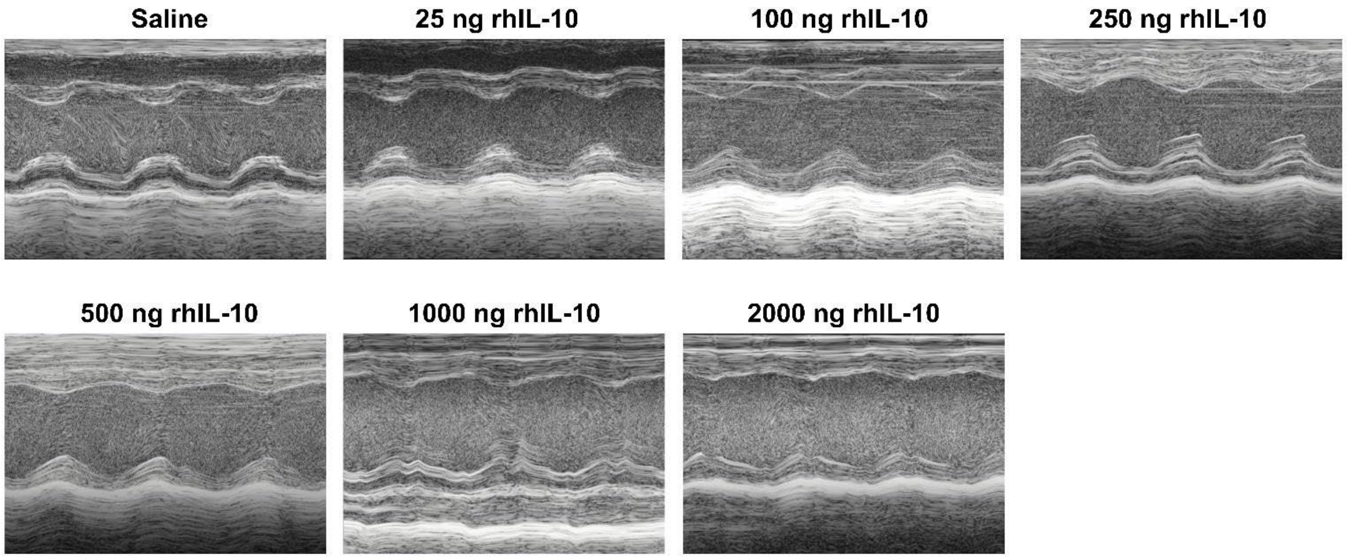

Supplemental Figure 3. Representative Echocardiographic Images at 5 days post-MI. Representative images extracted from echocardiographic recordings of 300 beats at 5 days post-MI for all tested groups, including the control (saline) and each dose group intramyocardially injected with rhIL-10 (25, 100, 250, 500, and 1000, and 2000 ng).

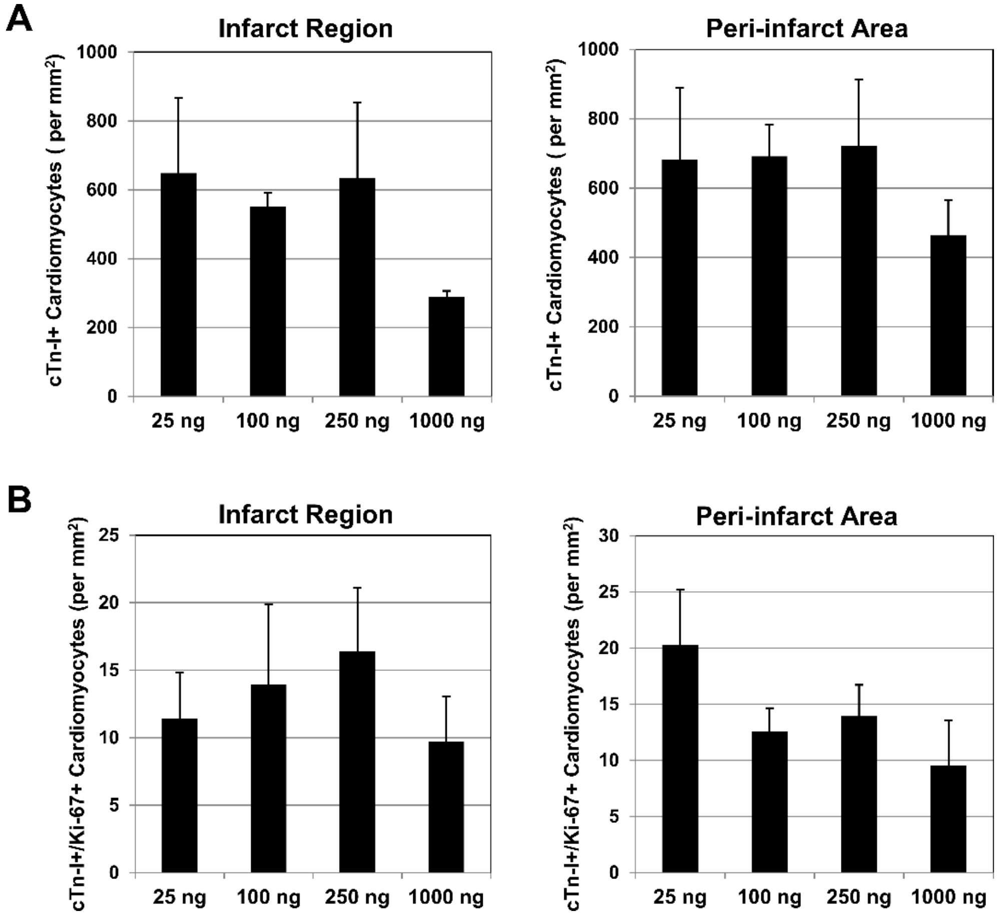

Supplemental Figure 4. Assessing the dose-dependent effects of intramyocardial IL-10 injection on long-term CM survival and proliferation post-MI. Infarcted mouse hearts acutely treated with different doses of hIL-10 via a single intramyocardial injection were harvested at 6 weeks post-MI, and serial cryosections were prepared and used for immunofluorescent staining. **(A)** Quantification of the residual CMs, labeled by a mature CM marker cTn-I, at the infarct and in peri-infarct areas. **(B)** Quantification of the proliferating CMs, identified by dual-positive signals of cTn-I and a cell proliferation marker Ki-67, at the infarct and in peri-infarct areas. No statistical significance was noted in all panels.
